# Clinical Characteristics of Frailty in Japanese Rheumatoid Arthritis Patients

**DOI:** 10.14283/jfa.2020.1

**Published:** 2020-01-10

**Authors:** Ichiro Yoshii, M. Kondo

**Affiliations:** 1 Yoshii Hospital, Shimanto-City, Kochi Prefecture, Japan; 2 Kondo Clinic for Rheumatology and Orthopedics, Fukuoka, Japan

**Keywords:** Rheumatoid Arthritis, frailty, aging, dementia, quality of life

## Abstract

**Objective:**

The relationship between clinical characteristics and frailty was investigated in rheumatoid arthritis (RA) patients >40 years old.

**Methods:**

RA patients followed for >1 year were interviewed and diagnosed as frail according to a 5-item frailty score index: (1) weight loss >2 kg within 6 months (WL); (2) slower gait speed (GS); (3) exercise less than once per week (EX); (4) decline in short-term memory (SM); and (5) general fatigue in the past 2 weeks (GF). The relationship between frailty status and background parameters was evaluated.

**Results:**

Among 739 subjects, frail patients comprised 221, pre-frail patients comprised 203, and robust comprised 315. The most common symptom in the Frailty group was GS, followed by SM, GF, EX, and WL, whereas the most common symptom in the Pre-frailty group was GS followed by SM, GF, WL, and EX. Frailty was significantly correlated with aging. Elderly onset rheumatoid arthritis, disease activity, serum C-reactive protein concentration, degree of joint deformity, activities in daily living (ADL), dementia treated, and glucocorticoid steroid administration demonstrated significant correlations with frailty status, although all factors also demonstrated significant correlation with aging. In addition, the EuroQol score (EQ5D) was significantly correlated with both aging and frailty.

**Conclusion:**

The results suggest that a remission state for disease activity, ADL, and dementia is correlated with frailty. The most common and primary symptom is GS. Elderly RA patients require careful attention for symptoms of frailty, which may damage the EQ5D score, specifically, the quality of life for RA patients.

## Introduction

As the number of elderly individuals is increasing worldwide, those with rheumatoid arthritis (RA) are increasingly presenting with age-specific problems. Frailty is one such problem and is a common but reversible decline in the physical status of elderly people. Frailty is defined as “excess vulnerability to stressors, with reduced ability to maintain or regain homeostasis after a destabilizing event.” Frailty is reflected in body composition and physical functioning and is associated with an increased risk of poor health outcomes and death (1). Frailty in the general population has been vigorously researched, and systemic treatment is now advocated (2). As with the elderly without arthritis, the increasing number of elderly individuals with RA requires increasing attention to the issue of frailty. However, there has as yet been little discussion in the literature about frailty specifically focused on those with RA (3, 4, 5, 6, 7, 8). The aim of this study was to investigate the characteristics of frailty in patients with RA, providing a framework for the development of coping strategies, and investigating the relationship between frailty and clinical parameters, particularly its correlation with aging.

## Materials and Methods

### Subject selection and frailty definition

From June to October in 2017, a total of 2193 RA outpatients diagnosed with 2010 ACR/EULAR classification criteria (9) aged 40 years and older who had been followed for more than 1 year had visited in each of the authors' main clinics. Among these, subjects were chosen and been interviewed for this study on first-come, first served basis. All patients were treated in accordance with the treat to target (T2T) strategy (10), meaning that each patient was treated with the goal of clinical remission in 6 months after baseline determination. Otherwise, the goal was low disease activity with shared decision between the physician and patient. Exclusion criteria were (1) admission experienced within 3 months, (2) bone fragility fracture history within 1 month, (3) cardiac disease administrated with beta blocker agent, and (4) dementia score with revised Hasegawa Dementia Scale (11) of ≤15 points because these patients are expected to be automatically classified as frailty due to physical and cognitive decline. A total of 739 patients were interviewed (586 women, 79.3%; 153 men, 20.7%). Using an established definition of frailty, individuals with three or more of the following physical deficits are classified as frail: (1) body mass index (BMI) ≤ 18.5; (2) low grip strength (measured with a handheld dynamometer and adjusted for sex and BMI); (3) severe fatigue (measured with the Multidimensional Assessment of Fatigue); (4) slow 4 m walking speed (adjusted for sex and height); and (5) low physical activity (measured using the International Physical Activity Questionnaire) (1). However, RA patient often has hand deformity that automatically leads grip force decline; thus, low grip strength has a risk to overlap frailty and RA natural status. Therefore, we abandoned the universal criteria. Then, we used the 5-item frailty score index developed by a Japanese physical therapist for elderly Japanese (12). This was developed by following 5852 individuals for 2 years and the association between higher frailty scores at baseline and long-term care insurance (LTCI) was analyzed. The tool is a simple questionnaire consisting of five categories. Frailty is diagnosed if three or more of the five items are positive, pre-frailty is diagnosed with two or one positive items, and robust is diagnosed with no items positive. The items consist of (1) weight loss of 2 to 3 kg in half a year (WL), (2) a reported slower gait speed (GS), (3) exercise habitat less than once per week (EX), (4) decline in short-term memory (SM), and (5) general fatigue in the preceding several weeks (GF). When patients were asked about these items, one point was assigned for each positive response and the total was defined as the frailty score. The criteria depend on patients' subjective feelings and tend to be too subjective; however, the most advantageous point in the criteria is the simplicity. The examiner can test in 5 minutes per subject and predict the necessity of LTCI services in Japan (12). Using this checklist, frailty in RA patients was investigated.

### Parameters measured

Other parameters assessed included sex, age at onset of RA and at the interview, the Health Assessment Questionnaire Disability Index (HAQ-DI) (13), the Simple Disease Activity Index (SDAI) (14) and its five components, the Sharp/van der Heijde Score (SHS) (15), the EuroQol 5 Dimension (EQ5D) (16) and its five categories, pain score measured with the visual analog scale (PS-VAS) (17), and drug administration status at the interview, and the number and type of comorbidities (N.Com) currently treated. Dementia was excluded from N.Com and was evaluated as an independent parameter because dementia overlaps SM. Every dementia patient somewhat treated with anti-dementia drug administration, such as donepezil hydrochloride, memantine, or rivastigmine. Data on the treatment of comorbidities, glucocorticoid steroid (GCS) administration, methotrexate (MTX) administration, biologic or targeted synthetic disease modifying anti-rheumatic drugs (b-/ts-DMARDs) administration were obtained from the patients' medical records. SHS was calculated by another radiologist who was trained in SHS calculation.

### Statistical analysis

Patients were classified by age in decades: 40s, 50s, 60s, 70s, 80s, and 90s. The number of patients, sex distribution, Steinbrocker Stage and Class of RA (18), and number of individuals diagnosed with frailty status as well as those with positive responses in each frailty category, average frailty score, average HAQ-DI score, the five categories in the EQ5D, SDAI, N.Com, and the percentage of patients with dementia treated were calculated for each age group. The results were compared among the age groups with a chi-squared test available for M x N for categorical variables or the Kruskal-Wallis test for continuous variables.

The association between the frailty status and other parameters was evaluated. In addition to the parameters used in the age group test, anti-cyclic citrullinated polypeptide antibodies (ACPA), rheumatoid factor (RF), SHS, GCS, MTX, and b-/ts-DMARDs administrated were evaluated. ANOVA with the Bonferroni correction was used for evaluations between the frailty states. Relationships between aging and the rest of parameters were evaluated for each with linear regression analysis to clarify residuals after the effect of aging was diminished. Relationship between the frailty score and residuals of the parameters and aging was then statistically evaluated using multivariate linear regression analysis.

In order to evaluate the relationship between the frailty status and age at onset and at current age, RA patients were divided into three categories in accordance with onset age and current age: patients with onset age ≥ 65 were classified as elderly onset rheumatoid arthritis (EORA), and the rest were classified as YORA. Those with current age ≥ 65 were further classified as oYORA, whereas the others were classified as yYORA. The number of each categorized cluster was calculated, and the distribution was statistically evaluated with M × N available chi-square tests. Statistical significance was set at <0.05.

All statistical procedures were performed using StatPlus:mac® (AnalystSoft Inc., Walnut, CA, USA).

## Results

### Comparison among age in decades

The results for the patients according to the age groups are shown in Table 1. Both the Stage and Class of RA and frailty scores were significantly higher among the older groups. Among the five frailty categories, the most frequently positive item was GS, followed in decreasing order by SM, GF, EX, and WL. Although only 5.2% of individuals in their 40s were diagnosed with frailty, the prevalence increased by decade to 100% for the seven patients in their nineties. The trend with age was the same for each of the five frailty categories. The HAQ-DI score was also significantly higher with older age. In the EQ5D, worse scores for transfer, instrumental ADL (IADL), and activity domains were significantly correlated with increasing age, with p < 1%. However, pain/discomfort did not significantly increase with age. SDAI had significant correlation with age, whereas the patient's global assessment and C-reactive protein (CRP) values were significantly correlated with age. N.Com as well as dementia demonstrated significant correlation with increasing age.

**Table 1:** Characteristics of patient's clinical background for generations

	40s	50s	60s	70s	80s	90s	p-value
Cases	58	89	135	260	190	7	
female	33 (56.9%)	74 (83.1%)	102 (75.6%)	211 (81.2%)	159 (84.2%)	7 (100%)	
Stage (I:II:III:IV)	40:7:6:5	55:19:12:3	67:31:22:15	87:77:25:71	52:67:8:70	1:3:1:2	0.96
Class (I:II:III:IV)	24:30:4:0	30:50:6:3	47:72:16:0	51:174:33:2	20:139:35:3	0:5:2:0	<1.00×10^−12^
Frailty: Pre-frailty: Robust	3:9:46	4: 22: 63	33:16:86	68: 103: 89	103: 54: 33	7:0:0	<1.00×10^−12^
average of the Frailty score	0.38	0.65	1.19	1.57	2.6	4.86	<1.00×10^−12^
Frailty category for WL	3 (5.2%)	6 (6.7%)	26 (19.3%)	47 (18.1%)	45 (23.7%)	6 (85.7%)	<1.00×10^−12^
Frailty category for GS	4 (6.9%)	18 (20.2%)	42 (31.1%)	122 (46.9%)	134 (70.5%)	7 (100%)	1.01×10^−11^
Frailty category for EX	5 (8.6%)	9 (10.1%)	29 (21.5%)	109 (41.9%)	117 (61.6%)	7 (100%)	<1.00×10^−12^
Frailty category for SM	7 (12.1%)	16 (18.0%)	38 (28.1%)	69 (26.5%)	88 (46.3%)	7 (100%)	<1.00×10^−12^
Frailty category for GF	3 (5.2%)	9 (10.1%)	25 (18.5%)	62 (23.8%)	94 (49.5%)	7 (100%)	<1.00×10^−12^
average HAQ-DI score	0.193	0.289	0.372	0.493	0.803	1.681	<1.00×10^−12^
EQ5D Transfer	1.17, 1–2	1.21, 1–2	1.33, 1–3	1.47, 1–3	1.86, 1–4	2, 1–4	9.84×10^−8^
EQ5D IADL	1.08, 1–2	1.16, 1–2	1.24, 1–3	1.28, 1–3	1.56, 1–3	2, 1–4	1.85×10^−5^
EQ5D Activity	1.13, 1–2	1.21, 1–2	1.31, 1–3	1.39, 1–3	1.7, 1–3	2, 1–3	9.50×10^−4^
EQ5D Pain/Discomfort	1.4, 1–2	1.5, 1–3	1.5, 1–3	1.51, 1–4	1.49, 1–4	2, 1–4	8.00×10^−4^
EQ5D Anxiety	1.08, 1–2	1.18, 1–2	1.21, 1–3	1.2, 1–3	1.24, 1–3	2, 1–4	0.64
SDAI	6.4, 1.4–31.5	7.3, 0.1–57.4	7.2, 0.1–65.8	5.5, 0.3–35.3	7.8, 0.1–40.3	9.1, 1.7–48.9	3.46×10^−07^
Number of Comorbidities by organ	2.6	3	3.2	3.6	2.5	1.5	7.47×10^−11^
Dementia treated	1.7%	3.4%	8.9%	25.0%	54.0%	75.0%	9.36×10^−4^


Patients with rheumatoid arthritis classified by age in decades: 40s, forties; 50s, fifties; 60s, sixties; 70s, seventies; 80s, eighties; 90s, nineties. Statistical evaluations compared variables among these age groups. The chi-squared test available for M × N was used to assess categorical variables. In other numerical parameters, statistical significance was evaluated with the Kruskal-Wallis test; Abbreviations: S.S., statistical significance; WL, weight loss of 2 to 3 kg in half a year; GS, slower gait speed reported; EX, exercise less than once a week; SM, decline in short-term memory; GF, general fatigue in preceding several weeks; HAQ-DI, Health Assessment Questionnaire Disability Index; EQ5D, EuroQol 5 dimensions; IADL, instrumental activity of daily living; TJC, tenderness joint count; SJC, swollen joint count; PGA, patient's global assessment; EGA, evaluator's global assessment; CRP, C-reactive protein; SDAI, Simplified Disease Activity Index; DAS28-CRP, 28-joint Disease Activity Score with CRP. In EQ5D categories and SDAI, mean value and range were shown.

### Prevalence of frailty items

**Figure 1:**
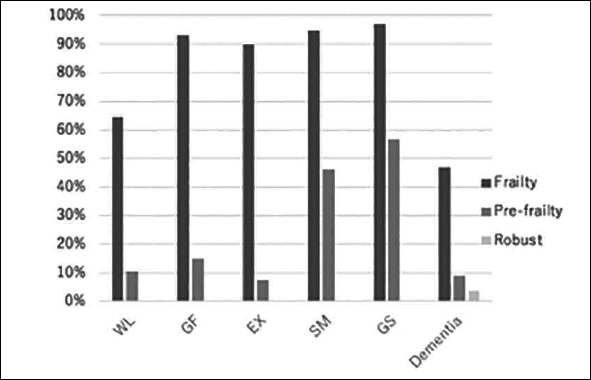
Positive rate of five items for frailty evaluation and dementia diagnosed. Most frequent subcategory is GS, followed by SM. This tendency is shown commonly by the Pre-frailty group. Positive rate in dementia is lower than the Frailty and the Pre-frailty groups for any items

Figure 1 shows the prevalence of the subcategories in the frailty status. The most frequent domain was GS followed by SM, GF, EX, and WL in the Frailty group and GS, SM, GF, WL, and EX in the Pre-frailty group in decreasing order. Prevalence of dementia treated demonstrated relatively lower values compare to the frailty items, that is more evidently shown until 60s; however, frailty followed by pre-frailty and robust, in the decreasing order of frequency, has the same pattern as the frailty items.

### Clinical characteristics of the frailty status

The clinical characteristics for each frailty status are shown in Table 2. The Frailty group had a higher proportion of women, oldest onset age, greater Clinical Disease Activity Score (CDAI), and greater CRP than the other two groups. Significant differences between the three frailty status groups were observed for the age at interview, RF, SHS, and HAQ-DI. In ACPA, PS-VAS, and N.com, the Frailty and Pre-frailty groups had significantly greater mean values than the Robust group. For drug use, GCS administration demonstrated significant higher ratio in the Frailty and Pre-frailty groups than in the Robust group, whereas MTX administration ratio demonstrated no significant differences between the groups. b-/ts-DMARD administration demonstrated significant lower ratio in the Robust group than in the other two groups.

**Table 2:** Comparison of clinical characteristics for Frailty, Pre-frailty and Robust groups

	Frailty	Pre-frailty	Robust	S.S. in the Frailty groups	S.S. after age correction	Correlation with the frailty score
female (%)	84.6	77.3	76.8	*	n.s.	n.s.
Age at onset	69.1	58.8	56.9	*		
Age at interview	79.4	67.5	63.3	**		p<0.01
ACPA	238.69	208.41	108.37	#	#	n.s.
RF	163.25	100.82	65.21	**	n.s.	n.s.
SHS	77.03	56.78	32.89	_**_	_*_	n.s.
HAQ-DI	0.795	0.428	0.235	_**_	_**_	p<0.01
CDAI	1.39	1.11	0.99	_*_	n.s.	n.s.
CRP	1.16	0.62	0.4	_*_	#	n.s.
PS-VAS	31.9	28.1	20.9	#	#	n.s.
N.Com	3.42	3.58	2.66	#	#	n.s.
Dementia treated	46.9	9.0	3.7	_*_	_*_	p<0.01
GCS administration (%)	62.3	56.7	36.5	#	_**_	p<0.01
MTX administration (%)	69.7	74.6	60.8	n.s.	n.s.	n.s.
b-/ts-DMARDs administration (%)	32.3	43.3	18.9	#	n.s.	n.s.


Abbreviations: HAQ-DI, Health Assessment Questionnaire Disability Index; CDAI, clinical disease activity score; CRP, C-reactive protein (mg/dl); PS-VAS, pain score with visual analog scale; N.Com, number of comorbidities treated; S.S., statistical significance compared between two of the groups with ANOVA; *, significantly greater in Frailty group than in the other groups; **, significantly greater in Frailty group than in the other groups, and significantly greater in Pre-frailty group than in Robust group; #, significantly greater in Frailty and Pre-frailty groups than in Robust group; Second right row shows statistical significance of correlation between parameters shown in most left row and ageing, and the most right row shows statistical significance of correlation between the parameters including age, and the frailty score with multivariate linear regression analysis.

In these parameters, ACPA, SHS, HAQ-DI, CRP, PS-VAS, N.Com, dementia treated, and GCS administration demonstrated significant correlation with aging, whereas after the effect of age was corrected, relationships were re-evaluated with MLR. Then, significant differences between the three frailty status groups were observed for HAQ-DI and GCS administration, whereas the Frailty group demonstrated a significantly higher value in SHS and higher prevalence in dementia treated than in the other two groups. The Robust group demonstrated a significantly lower value than the Pre-frailty and Frailty groups in ACPA, CRP, PS-VAS, and N.Com even after the effect of age was corrected.

The correlation with the frailty score of these parameters demonstrated statistical significance of <1% in age at interview, HAQ-DI, dementia treated, and GCS administration.

### Relationship between EQ5D categories and the frailty status

**Figure 2:**
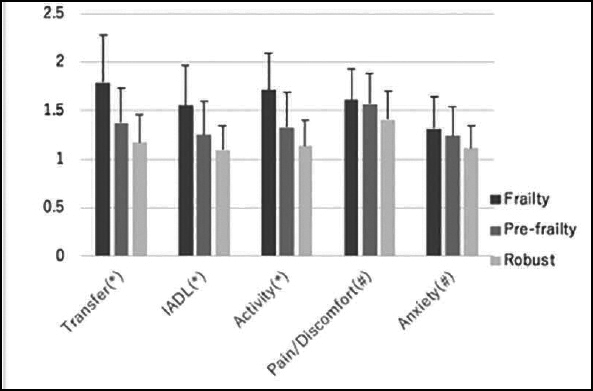
Positive rate of five items for frailty evaluation and dementia diagnosed. Most frequent subcategory is GS, followed by SM. This tendency is shown commonly by the Pre-frailty group. Positive rate in dementia is lower than the Frailty and the Pre-frailty groups for any items

In the EQ5D subdimensions, transfer, IADL, and activity were significantly higher in the Frailty group than in the other two groups. These items were higher in the Pre-frailty group than in the Robust group. There was no significant difference between the Frailty and Pre-frailty groups for pain/discomfort and anxiety, but the two groups had significantly higher results than the Robust group (Figure 2).

### Prevalence of the frailty status in according to onset age groups

Average age at interview was 79.9, 68.7, and 49.8 years for EORA, oYORA, and yYORA, respectively, and disease duration at interview was 9.2, 16.5, and 7.8 years respectively. These parameters demonstrated significant difference among all groups. The prevalence of frailty was the most frequently seen in the EORA patient group than in the other groups, although there was no significant difference between the EORA and oYORA group when prevalence of frailty and pre-frailty were added up together (Table 3).

**Table 3:** Average age, disease duration of EORA, oYORA, and yYORA, and case numbers of each group in the frailty status

	EORA	oYORA	yYORA	Total
average age at interview	79.9	68.7	49.8	71.3
disease duration at interview	9.2	16.5	7.8	10.6
Frailty	169 (42.4%)	48 (23.3%)	4 (3.0%)	221
Pre-frailty	92 (23.1%)	75 (36.4%)	36 (26.9%)	203
Robust	138 (34.6%)	83 (40.3%)	94 (70.1%)	315
Total	399	206	134	739


Abbreviations; EORA, elderly onset rheumatoid arthritis patient; oYORA, youngly onset old rheumatoid arthritis patient; yYORA, youngly onset young rheumatoid arthritis patient. Definition: EORA, onset age>=65; oYORA, onset age<65 and age at interview>=65; yYORA, onset age<65 and age at interview<65.

## Discussion

Patients with RA have an increasingly greater life expectancy because of the introduction of new drugs that improve control of the disease. However, this also means that more patients may be subject to frailty as they age. Caring for such patients therefore raises frailty as an additional challenge (19).

In this study, we attempted to clarify the characteristics associated with the frailty status and the frailty score, as well as the relationship between diagnosis or the frailty status and various clinical parameters such as aging, ADL, quality of life, state of disease activity, comorbidities, and dementia. We were afraid that disease activity would confound the frailty status; therefore, patients who had been treated for >1 year were picked up. Average disease activity calculated with SDAI in all generations were <ten, which means low disease activity level. As is generally accepted, aging has a close correlation with the Frailty status. In a comparison among generations, the prevalence of frailty increased with age in addition to the average frailty score. However, a remarkable finding in this study is that nearly one-fourth of RA patients in their sixties were included in the Frailty group. The prevalence was not significantly higher in the seventies age group, but the proportion of Pre-frailty status patients increased. Compared with Yamada's report, every item for the diagnosis of frailty in patients aged >60 years in the present study demonstrated obviously higher values of positive ratio (Table 4) (12). These results suggest that physical instability in RA patients begins at a younger age than other populations reported previously (19, 20). This can result from long-lasting inflammation, leading to joint deformity, and fatigue or psychological burden (21). Even though RA treatment has been improved and disease activity is well controlled, these problems remain unsolved. The results in this study suggest that physical and psychological care is necessary for RA patients aged 60 and above to prevent frailty.

**Table 4:** Compare to Yamada's study for prevalence of five items for frailty

	Yamada	Present study (in no younger than 60s)	Present study (crude)
WL	12.6%	20.9%	18.0%
GS	54.3%	51.5%	44.2%
EX	19.5%	44.3%	37.3%
SM	7.4%	34.1%	30.4%
GF	20.4%	30.7%	26.3%


Abbreviations: WL, weight loss of 2 to 3 kg in half a year; GS, slower gait speed reported; EX, exercise less than once a week; SM, decline in short-term memory; GF, general fatigue in preceding several weeks.

To provide further evidence, we evaluated the patient numbers in the EORA, oYORA, and yYORA frailty status groups. The results showed that EORA was significantly more common than YORA in the Frailty group, whereas EORA and oYORA were significantly more common in the Pre-frailty group than in the control group, indicating that elderly RA patients have a strong risk of frailty.

Gait status in particular seems to be the most important factor, because the most frequent factor in the five categories of frailty for the sixties age group was GS. This finding indicates that GS and gait instability are to be evaluated in patients in their sixties. GS loss was also the most popular symptom in the Frailty group. Gait ability evaluation is therefore essential for good control of physical stability in RA patients. A decline in ADL, particularly locomotive function, is closely related to aging. We found that GS was the most frequently reported symptom of frailty, suggesting that daily exercise is important to avoid frailty, as has been recommended by other investigators (22, 23). RA patients have a weak grip force or upper extremity weakness due to joint damage (24); however, upper extremity weakness does not always figure into frailty assessment. We recommend that more attention should be paid to locomotive functions for RA patients.

The EQ5D Activity category, a reflection of ADL functioning, significantly correlated with both the diagnosis of frailty and the frailty score. These results suggested that quality of life is strongly associated with frailty. Therefore, we conclude that further investigation addressing ADL to prevent frailty is necessary, which may lead the maintenance of quality of life.

It was also clear from this study that dementia is strongly correlated with the frailty status as well as aging. Frailty, aging, and dementia are likely to go hand-in-hand.

In comparison with the clinical background of RA for the frailty status, the sex, age at onset and at interview, ACPA, RF, SHS, HAQ-DI, CDAI, CRP, PS-VAS, N.com, dementia treated, GCS administration, MTX administration, and all parameters except b-/ts-DMARDs administration were investigated and demonstrated that significant factors that correlated with the frailty status significantly were ACPA, SHS, CRP, PS-VAS, N.Com, and GCS administration after aging was corrected, whereas age at interview, HAQ-DI, dementia treated, and GCS administration demonstrated significant correlation with the frailty score. These results suggested that such is the deepness of aging reflection in the frailty status. At the same time, frailty status is influenced by various factors, such as ACPA titer, joint deformation, ADL represented by the HAQ score, inflammation status represented with CRP, number of comorbidities, and GCS administration. These may be independent risk factors of the frailty status besides aging. Patients with the above risk factors tend to develop frailty; therefore, more careful attention is warranted. In these factors, the HAQ score, whether dementia treated or GCS administered, are the noticeable parameters for early detection of frailty.

One notably important factor is GCS administration, which declined the frailty score as shown in Table 2. Although short GCS administration is recommended in the EULAR recommendation for RA in 2016 (25), GCS administration should be considered with discretion.

Increased CRP levels were significantly correlated with the frailty status. Inflammation as reflected in serum markers has been identified as indicating increased risk of frailty in patients with RA (26). Our results are consistent with this finding, emphasizing the importance of controlling inflammation in patients with RA. Monitoring CRP levels is necessary not only to manage disease activity but also for addressing frailty.

The number of organ systems involved with comorbidities was significantly correlated with the frailty status. Prevalence of comorbidities showed obviously higher ratio than that obtained in Yamada's report (12). However, no significant difference in frequency was demonstrated among generations except with dementia (Supplemental Table). These results were rather surprising as we expected the musculoskeletal and cardiovascular systems to be most strongly associated with frailty since disorders in those two systems can affect physical function. However, there was no correlation between musculoskeletal system disorders and frailty in this study. These findings may be because of the nature of the Frailty questionnaire. Three of the 5-item frailty score questions are subjective. Patients might indicate that they were not bothered by slower GS, short-term memory loss, or general fatigue and therefore would not be considered frail, despite objective evidence of a number of comorbidities. This may have confounded analysis of the association between comorbidities and frailty in accordance with the frailty score.

There are several major limitations to be considered when interpreting our results. (1) The cross-sectional study design did not allow for longitudinal observations. (2) The presence of dementia was determined on the basis of whether a patient was being treated for it, not on the basis of a diagnosis or stage of dementia. (3) The effects of other potential confounding factors — such as sex, muscle power, osteoporosis, polypharmacy, ethnicity, RA disease duration, and joint destruction — were not assessed.

There is no article that has investigated regarding frailty management for RA patients in our investigation scope; however, some cohort studies have described regarding frailty management. Of these, Leng et al. have reported that higher serum interleukin 6 (IL-6) levels and lower hemoglobin levels were observed in the frailty subjects. They concluded that potential increase in chronic inflammation state is related to the frailty status (26, 27). These results coincide with the results of our study that CRP level correlates with the frailty status. The Asia-Pacific Clinical Practice Guidelines for the Management of Frailty strongly recommends physical activity with a resistance training component along with various other physical activities, including balance training (28). These trainings control serum IL-6, one of the myokines (29) and would be a hint to manage frailty.

We investigated whether patients with RA had a frailty risk and the factors potentially associated with the frailty score. Although many factors, such as aging, being treated for dementia, GCS administration, ADL decline, especially particularly gait function or speed, and number of comorbidities, are confounded, RA patients may have a risk of falling into frailty in a younger age than those who are not suffering from RA. Elderly RA patients ≥ 60 have a strong risk of frailty or pre-frailty; therefore, controlling the physical functions of these patients is essential to avoid frailty.

*Compliance with Ethical Standards:* This study was conducted in compliance with the Ethical Guidelines for Medical and Health Research Involving Human Subjects of Japan under the Declaration of Helsinki. Protocol and consent forms were approved by the Ethics Committee of the institutions to which the authors belonged, which comprises director, plural physicians, and co-medical managers. Patients and their families were informed that personal information would be anonymous and used only for analysis before they signed the consent forms.

*Conflict of Interest statement:* All authors, Ichiro Yoshii and Masakazu Kondo, and their families have no conflicts of interest directly or indirectly relevant to the content of this manuscript. Furthermore, all authors and their families have no financial support or other benefits from commercial sources for the work reported in this manuscript, or any other financial interests or grants, which could create a potential conflict of interest.

## Electronic supplementary material

Supplemental table: Total number of comorbidities for organs and prevalence in percentage by age in decades

## References

[1] Fried LP, Tangen CM, Walston J, Cardiovascular Health Study Collaborative Research G (2001). Frailty in older adults: evidence for a phenotype. J Gerontol A.

[2] Fried LP, Darer D, Walston J, Cassel CK, Leipzig R, Cohen HA, Larson EB, Meier DE (2003). Geriatric Medicine.

[3] Andrews J, Wahl E, Schmajuk G, Yelin E, Katz P (2016). THU0096 Serum Inflammation Identifies Increased Risk of Frailty in Rheumatoid Arthritis. Ann Rheum Dis suppl.2.

[4] Andrews JS, Trupin L, Yelin EH, Hough CL, Covinsky KE, Katz PP (2017). Frailty and reduced physical function go hand in hand in adults with rheumatoid arthritis: a US observational cohort study. Clin Rheumatol.

[5] Li G, Li X, Cesta A, Lau A, Bombardier C, Adachi JD (2017). Frailty and risk of fractures in patients with rheumatoid arthritis: Data from the Ontario Best Practices Research Initiative (OBRI). Arthritis Rheumatol.

[6] Andrews J, Covinsky K, Hough C, Trupin L, Yelin EH, Katz PP (2016). Frailty is associated with decreased physical function in adults with rheumatoid arthritis. Arthritis Rheumatol.

[7] Salaffi F, Di Carlo M, Farah S, Di Donato E, Carotti M (2019). Prevalence of frailty and its associated factors in patients with rheumatoid arthritis: a cross-sectional analysis. Clin Rheumatol.

[8] Haider S, Grabovac I, Berner C, Lamprecht T, Fenzl KH, Erlacher L, et al. Frailty in seropositive rheumatoid arthritis patients of working age: a cross-sectional study. Clin Exp Rheumatol (in press) 30557129

[9] Aletaha D, Neogi T, Silman AJ (2010). 2010 Rheumatoid arthritis classification criteria: An American College of Rheumatology/European League Against Rheumatism collaborative initiative. Arthititis Rheumatol.

[10] Smolen JS, Aletaha D, Bijlsma JWJ, T2T Expert Committee (2010). Treating rheumatoid arthritis to target: recommendations of an international task force. Ann Rheum Dis.

[11] Imai Y, Hasegawa K (1994). The revised Hasegawa's Dementia Scale (HDS-R)-Evaluation of its usefulness as a screening test for dementia. J Hong Kong Coll Psychiatr.

[12] Yamada M, Arai H (2015). Predictive value of frailty scores for healthy life expectancy in community-dwelling older Japanese adults. J Am Med Dir Assoc.

[13] Fries JF, Spitz P, Kraines RG, Holman HR (1980). Measurement of patient outcome in arthritis. Arthritis Rheum.

[14] Aletaha D, Smolen JS (2005). The Simplified Disease Activity Index (SDAI) and the Clinical Disease Activity Index (CDAI): a review of their usefulness and validity in rheumatoid arthritis. Clin Exp Rheumatol.

[15] Van der Heijde D, Dankert T, Nieman F, Rau R, Boers M (1999). Reliability and sensitivity to change of a simplification of the Sharp/van der Heijde radiological assessment in rheumatoid arthritis. Rheumatol.

[16] Hurst NP, Kind P, Ruta D, Hunter M, Stubbings A (1997). Measuring health-related quality of life in rheumatoid arthritis: validity, responsiveness and reliability of EuroQol (EQ-5D). Rheumatology.

[17] Hawker GA, Mian S, Kendzerska T, French M (2011). Measures of adult pain: Visual Analog Scale for Pain (VAS Pain), Numeric Rating Scale for Pain (NRS Pain), McGill Pain Questionnaire (MPQ), Short-Form McGill Pain Questionnaire (SF-MPQ), Chronic Pain Grade Scale (CPGS), Short Form-36 Bodily Pain Scale (SF-36 BPS), and Measure of Intermittent and Constant Osteoarthritis Pain (ICOAP). Arthritis Care & Research (suppl11).

[18] Steinbrocker O, Traeger CH, Batterman RC (1949). Therapeutic criteria in rheumatoid arthritis. JAMA.

[19] Kojima G, Iliffe S, Taniguchi Y, Shimada H, Rakugi H, Walters L (2017). Prevalence of frailty in Japan: A systematic review and meta-analysis. J Epidemiol.

[20] Collard RM, Boter H, Schoevers RA, Oude Voshaar RC (2012). Prevalence of Frailty in Community-Dwelling Older Persons: A Systematic Review. J Am Geriatr Soc.

[21] Brown AK, Conaghan PG, Karim Z (2008). An explanation for the apparent dissociation between clinical remission and continued structural deterioration in rheumatoid arthritis. Arthritis Rheum.

[22] Brown M, Sinacore DR, Ehsani AA, Binder EF, Holloszy JO, Kohrt WM (2000). Low-intensity exercise as a modifier of physical frailty in older adults. Arch Phys Med Rehabil.

[23] Fiatarone MA, O'Neill EF, Ryan ND (1994). Exercise training and nutritional supplementation for physical frailty in very elderly people. N Engl J Med.

[24] Berner C, Erlacher L, Quittan M, Fenzl KH, Dorner TE (2017). Workability and muscle strength in patients with seropositive rheumatoid arthritis: Survey Study Protocol. JMTR Res Protoc.

[25] Smolen JS, Landewé R, Bijlsma J (2017). EULAR recommendations for the management of rheumatoid arthritis with synthetic and biological disease-modifying antirheumatic drugs: 2016 update. Ann Rheum Dis.

[26] Andrews JS, Wahl ER, Schmajuk G, Yelin EH, Katz PP (2015). Serum inflammation identifies increased risk of frailty in rheumatoid arthritis. Arthritis Rheumatol.

[27] Leng S, Chaves P, Koenig K, Walston J (2002). Serum interleukin-6 and hemoglobin as physiological correlates in the geriatric syndrome of frailty: a pilot study. J Am Geriatr Soc.

[28] Dent E, Lien C, Lim WS (2017). The Asia-Pacific clinical practice guidelines for the management of frailty. J Am Med Dir Assoc.

[29] Pedersen BK, Fischer CP (2007). Benefical health effects of exercise — the role of IL-6 as a myokine. Trends Pharmacol Sci.

