# Supplementary material for: Clinical Characteristics of Frailty in Japanese Rheumatoid Arthritis Patients
Source: J Frailty Aging. 2020 Jan 10;9(3):158–64. doi: 10.14283/jfa.2020.1 (PMC12275781; doi:10.14283/jfa.2020.1)
Supplement: Supplementary file 1 — Supplemental table: Total number of comorbidities for organs and prevalence in percentage by age in decades [file mmc1.docx]

| Supplemental table: Total number of comorbidities for organs and prevalence in percentage by age in decades | | | | | | | |
| --- | --- | --- | --- | --- | --- | --- | --- |
|  | 40s | 50s | 60s | 70s | 80s | 90s | S.S. |
| Comorbidities by organ | 2.6 | 3 | 3.2 | 3.6 | 2.5 | 1.5 | n.s. |
| Musculoskeletal | 58.60% | 60.70% | 68.10% | 73.90% | 49.20% | 50.00% | n.s. |
| Neural | 8.60% | 22.50% | 20.00% | 27.20% | 25.40% | 0.00% | n.s. |
| Respiratory | 37.90% | 41.60% | 43.00% | 45.70% | 30.20% | 0.00% | n.s. |
| Cardiovascular | 8.60% | 22.50% | 29.60% | 42.40% | 33.30% | 25.00% | n.s. |
| Immune | 19.00% | 15.70% | 10.40% | 15.20% | 3.20% | 0.00% | n.s. |
| Endocrine & Metabolism | 36.20% | 37.10% | 35.60% | 38.00% | 22.20% | 25.00% | n.s. |
| Skin | 15.50% | 20.20% | 25.90% | 33.70% | 20.60% | 0.00% | n.s. |
| Urogenital | 1.70% | 4.50% | 4.40% | 8.70% | 7.90% | 0.00% | n.s. |
| Digestive | 41.40% | 48.30% | 47.40% | 47.80% | 36.50% | 0.00% | n.s. |
| Neoplasm | 1.70% | 0.00% | 4.40% | 2.20% | 1.60% | 0.00% | n.s. |
| Others | 25.90% | 24.70% | 27.40% | 22.80% | 20.60% | 50.00% | n.s. |
| Dementia treated | 1.70% | 3.40% | 8.90% | 25.00% | 54.00% | 75.00% | # |
| Statistical evaluations compared variables among these age groups. The chi-squared test available for M x N was used to assess categorical variables. Abbreviations: 40s, forties ; 50s, fifties ; 60s, sixties ; 70s, seventies ; 80s, eighties ; 90s, nineties ; S.S., statistical significance ; n.s., not significant ; #, significantly increased as ages get older within 5%. | | | | | | | |
|  |  |  |  |  |  |  |  |
|  |  |  |  |  |  |  |  |
|  |  |  |  |  |  |  |  |
|  |  |  |  |  |  |  |  |
